# Newborn screening for Morquio disease and other lysosomal storage diseases: results from the 8-plex assay for 70,000 newborns

**DOI:** 10.1186/s13023-020-1322-z

**Published:** 2020-02-03

**Authors:** Yin-Hsiu Chien, Ni-Chung Lee, Pin-Wen Chen, Hui-Ying Yeh, Michael H. Gelb, Pao-Chin Chiu, Shao-Yin Chu, Chen-Hao Lee, An-Ru Lee, Wuh-Liang Hwu

**Affiliations:** 1 Department of Medical Genetics, National Taiwan University Hospital, Taipei, Taiwan; 2 Department of Pediatrics, National Taiwan University Hospital, and National Taiwan University College of Medicine, Taipei, Taiwan; 3 Department of Chemistry, University of Washington, Seattle, WA 98195 USA; 4 Department of Pediatrics, Kaohsiung Veterans General Hospital, Kaohsiung, Taiwan; 5 Department of Pediatrics, Buddhist Tzu-Chi General Hospital, Hualien, Taiwan; 6 Department of Pediatrics, E-DA Hospital, Kaohsiung City, Taiwan

**Keywords:** Lysosomal storage disease, Mucopolysaccharidosis, Multiplex newborn screening, Morquio disease, MPS4A, Tandem mass spectrometry

## Abstract

**Background:**

The necessity of early treatment for lysosomal storage diseases (LSDs) has triggered the development of newborn screening for LSDs in recent years. Here we report the first 70,000 newborns screened for Mucopolysaccharidosis (MPS) type 4A (Morquio syndrome) and other LSDs by an 8-plex assay including the original 4-plex LSD screening tandem mass spectrometry (MS/MS) assay for Pompe disease, Fabry disease, Gaucher disease, and MPS I disease.

**Methods:**

The additional reaction for MPS II, MPS 3B, MPS 4A, and MPS 6 enzymes was performed separately from the 4-plex reaction. The two reactions were quenched and extracted, then combined before carrying out a single 2-min UPLC-MS/MS analysis.

**Results:**

From Mar. 2018 to Apr. 2019, 73,743 newborns were screened with the 8-plex LSD screening assay. The 8-plex assay revealed a better analytical precision than the previous 4-plex assay possibly because the 8-plex was carried out using UPLC-MS/MS. Six newborns were found to have low MPS-4A enzyme (N-acetylgalactosamine-6-sulfatase) activity and biallelic *GALNS* pathogenic mutations *in trans*; these patients are presumably affected with MPS4A, making an incidence of one in 12,291 (95% confident interval (CI): 5633-26,817). One mutation, c.857C > T (p.T286 M) of the *GALNS* gene, accounted 5 of the 12 mutated alleles. These newborns had immature vertebral bodies at 1 month of age, and one case was treated with elosulfase alfa 2 mg/kg/week starting from 4 months of age. Among other MPSs screened, one case of MPS I, 3 cases of MPS II, and 3 cases of MPS 3B were detected. One case of mucolipidosis type III was also diagnosed. In conjunction with another 9 patients of Pompe disease, Gaucher disease, and classical Fabry disease, making an incidence of LSDs as one in 3206 newborns (95% CI: 2137 - 4811). The one with infantile-onset Pompe disease and the one with Gaucher disease were treated since the age of 8 days and 41 days respectively.

**Conclusions:**

Routine newborn screening of MPS 4A and other LSDs were made possible by the 8-plex LSD screening assay. However, detailed phenotype prediction and the time to start treatment will need further elucidation.

## Background

Lysosomal storage diseases (LSDs) are caused by a deficiency of one of the lysosomal acid hydrolases. Nowadays, several LSDs can be treatable with either enzyme replacement therapy (ERT), pharmaceutical chaperones, substrate reduction, or hematopoietic stem cell transplantation. Because LSDs often lead to irreversible damage to the cells and tissues, such as damages to the skeletal muscle (eq. in Pompe disease), bones (eq. in a few types of mucopolysaccharidosis (MPSs)), and the nervous system (eq. in most types of neuropathic LSDs), these diseases can be devastating at the time of clinical recognition of symptoms. Therefore, the necessity of early treatment has been proposed for many of the LSDs.

Newborn screening for LSDs enables early initiation of treatment, and a multiplex platform is needed for screening several LSDs simultaneously. Nowadays, there are two major ways to perform a multiplex assay, the digital microfluidic fluorometric method, and the tandem mass spectrometry (MS/MS) methods [1–4]. Our initial LSD screening assays for Pompe and Fabry diseases were conducted using fluorescence substrates [5], with little potential for further multiplexing. Therefore, we changed to the 4-plex LSD screening tandem mass spectrometry (MS/MS) assay for Pompe disease, Fabry disease, Gaucher disease, and MPS I [6]. We recently updated the method into an 8-plex assay, with the addition of screening for MPS II, MPS 3B, MPS 4A, and MPS 6. The method has been validated by a pilot study in Washington state, USA, using de-identified dried blood spots [7].

Mucopolysaccharidosis type IVA (MPS 4A), also known as Morquio (Morquio-Brailsford) syndrome results from the accumulation of keratan sulfate (KS) and chondroitin-6-sulfate (C6S), whereas the primary cause is mutations in the gene encoding N-acetyl-galactosamine-6-sulfatase (GALNS). The substrates are stored primarily in the cartilage and its extracellular matrix (ECM), leading to a direct impact on bone development and successive systemic skeletal spondylepiphyseal dysplasia. Enzyme replacement therapy (ERT) with elosulfase alfa is the only approved therapy. Considering the irreversible damage usually seen in patients with MPS 4A, International management guidelines recommend that elosulfase alfa treatment be implemented as soon as the diagnosis of MPS 4A has been confirmed [8]. Although there is limited experience in the presymptomatic treatment of MPS 4A, early treatment, based on the progressive nature of the disease, is likely to be beneficial. Therefore, we decided to carry out the first large scale prospective newborn screening of MPS 4A, together with other LSDs using the 8-plex assay, and the results are reported here.

## Materials and methods

The Newborn Screening Center at the National Taiwan University Hospital (NTUH) performs routine newborn screening for approximately 35% of newborns in Taiwan or 70,000 newborns per year. The center initiated a pilot screening program for Pompe disease in 2005 and Fabry disease in 2006. In 2008, Pompe disease screening was added to the regular screening items. In 2015, we started the four-plex assay: Pompe disease, MPS I, Gaucher disease, and Fabry disease. Parents of newborns needed to give consent for the LSD multiplex assay [6]. In 2018, we added 4 more conditions in this LSD multiplex assay. Newborns with screening positives were referred to NTUH for confirmatory testing.

### Screening assay

Enzyme substrates, internal standards, and assay buffer were purchased from PerkinElmer (Turku, Finland). Newborn DBSs were punched as 3 mm each into duplicate 96-well microtiter plates using a Wallac DBS Puncher. One plate received an LSD quadruplex assay cocktail associated with Pompe, Fabry, Gaucher, and MPS I diseases, while the other plate received assay cocktail of MPS II, MPS 3B, MPS 4A, and MPS 6. Plates were sealed with aluminum sealing film for 16 h incubation at 37 °C with orbital shaking. After overnight incubation, the enzyme reaction was quenched with 100 μL of 1:1 methanol/ethyl acetate solution. The products and internal standards were separated from the buffer by liquid-liquid extraction using 400 μL of ethyl acetate and 200 μL of purified water for the LSD quadruplex assay, and 400 μL of ethyl acetate and 200 μL of 0.5 M NaCl in water for the MPS assay. Aliquots 200 μL of the ethyl acetate phase from duplicate wells were combined into a single well, evaporated and reconstituted in 45% acetonitrile with 0.1% formic acid for UPLC-MS/MS analysis.

### UPLC-MS/MS analysis

UPLC-MS/MS was performed on a XEVO TQD triple-quad mass spectrometer (Waters, Milford, MA) in positive ion mode. Aliquots of the samples (5 μL) were injected into an analytical column (ACQUITY UPLC CSH C18; 2.1 × 50 mm, 1.7 μm) with a gradient separation by mobile phase A (30% acetnitrile/70% water with 0.1% formic acid) and mobile phase B (50% acetonitrile/50% isopropanol with 0.1% formic acid) at a flow rate of 0.8 mL/min at 55 °C under the following gradient conditions: linear gradient from 1 to 70% B from 0 to 1.0 min; linear gradient from 70 to 75% B from 1.0 to 1.5 min; decreased to 1% B and re-equilibrated for 0.5 min. Data were collected during 1.6 min of sample infusion (Additional file 4: Figure S1). The total running time for one plate was approximately 3 h. The enzyme activity was calculated in μM/h from the ratio of isotopic-substituted enzymatic product to internal standards.

### Algorithm

For Pompe disease, the algorithm we used was mentioned in our previous publications [6], i.e., both the first screening cutoff and the critical cutoff were used to determine whether to proceed to the second tier assay or whether to refer for a diagnostic evaluation immediately. The diagnostic evaluation includes bringing in the baby to our hospital for a complete cardiac and physical checkup, lymphocyte GAA activity measurement, urine glucose tetrasaccharide (Glc4) measurement, and GAA mutation analysis if necessary.

For the other conditions, only the screening cutoffs were applied (Additional file 1: Table S1). For newborns who showed a first-round enzymatic activity below the cutoff, a second sample was requested (recall). Newborns with an abnormal recall result were referred to our hospitals for a diagnosis testing. The tests included leukocyte enzyme activity measurement, mutation analysis, and biomarker measurements. The respective biomarkers included plasma lysoglobotriaosylceramide (LysoGb3) for Fabry disease, plasma glucosylsphingosine (LysoGb1) for Gaucher disease, total urine glycosaminoglycans (GAGs) by the dimethylmethylene blue (DMB) assay for MPSs, and urine keratan sulfate (KS) by LC-MSMS for MPS 4A.

This study was approved by the institutional review board (201906053RINB).

## Results

### Total incidence

Between Mar. 2018 and Apr. 2019, 73,743 newborns were tested with the 8-plex MS/MS assay. In total, 99.3% of the newborns did not display any enzymatic activities below the cutoff values (values given in Additional file 1). Results from eighty-one newborns (0.1%) were regarded as unsatisfactory due to low levels of multiple enzyme activities, and a second dried blood spot sample was requested for these newborns. For newborns that showed a single enzymatic activity below the cutoff (*n* = 361, 0.5%) (except for Pompe disease), a second sample was requested. The recall rate varied from 0.01 to 0.24% (Table 1). For newborns with GAA deficiency in the first round (*n* = 157, or 0.2%), 3 (0.004%) met the critical cutoff and went directly to the confirmatory checkup. In addition, 154 (0.2%) met the borderline cut off and went to a 2nd tier assay, and 3 were subsequently sent for a confirmatory checkup. In total, only 6 (0.008%) newborns were screen positive for Pompe disease and went for the diagnostic testing, at the age of 6–9 days.

**Table 1:** Incidence of the eight conditions screened by the 8-plex assay

Condition	Numbers of DBS samples failed in the 1st screen	Screen positives	Affected Numbers	Phenotype	Incidence (1 in) (95% CI)
Pompe	–	6	4	1 IOPD, 3 LOPD	18,436 (7170-47,407)
Fabry	5 (0.01%)	4	4	2 classic, 2 suspected classic	18,436 (7170-47,407)
Gaucher	27 (0.04%)	9	1	type 3	73,743 (13,018-417,749)
MPS I	178 (0.24%)	10	1	suspected severe	73,743 (13,018-417,749)
MPS II	56 (0.08%)	32	3^a^	suspected mild	24,581 (8360-72,277)
MPS 3B	14 (0.02%)	3	3		24,581 (8360-72,277)
MPS 4A	70 (0.09%)	12	6		12,291 (5633-26,817)
MPS 6	11 (0.01%)	0	0		< 73,743
ML	–	–	1	suspect ML-III	73,743 (13,018-417,749)
Total MPSs			14		5267 (3138-8842)
Total			23		3206 (2137-4811)

*IOPD* infantile-onset Pompe disease, *LOPD* later-onset Pompe disease, ^a^another 29 have benign variants, *ML* mucolipidosis

In summary, 23 newborns were regarded as affected patients, including early-onset and genotypes associating with the late-onset phenotypes (Table 1). The one with infantile-onset Pompe disease was treated with recombinant alpha-glucosidase since the age of 8 days. The one with Gaucher disease was treated since the age of 41 days due to thrombocytopenia [9]. Others were not on treatment yet. One newborn with high MPS II activity (6-fold of the mean normal I2S) and MPS 3B (6-fold of the normal NAGLU mean) was found. Confirmation analysis led to a diagnosis of mucolipidosis, probably type III. The overall incidence for all tested LSDs was one in 3206 newborns (95% confidence interval (CI): 1 in 2137 to 4811). The incidence for those treatable MPS diseases was one in 7374 (95% CI: 1 in 4006 to 13,575).

Data for biomarkers and genotypes from confirmed cases except MPS 4A were listed in Table 2. Newborns with the early onset forms of Pompe disease, Gaucher disease, and classic type of Fabry disease had high biomarker levels. Newborns with potential later-onset Pompe disease (*GAA* c.[752C > T; 761C > T] (p.[S251 L; S254 L]), *GAA* c.546 + 5 G > T) [10] or probably late-onset Fabry disease (*GLA* c.1078G > T (p.G360C)) [5] had normal or borderline normal biomarkers. Although newborns with MPS I and MPS II have novel genotypes, all MPS patients showed only slightly elevated urinary GAGs above the normal reference range, except the MPS 3B patients show the highest levels of urinary GAGs.

**Table 2:** Genotypes and biomarkers level of patients identified in this study except for MPS 4A

Condition	Genotype	Biomarkers
Pompe	c.2024_2026del (p.N675del)/c.2040 + 1G > T^a^	45.03
	c.[752C > T; 761C > T] (p.[S251 L; S254 L])/c.[752C > T; 761C > T] (p.[S251 L; S254 L])	20.46
	c.[1935C > A;1726G > A] (p.[D645E; G576S])/c.546 + 5 G > T	18.61
	c.1091C > T (p.P364L)/c.1843G > A (p.G615R)	8.48
Fabry	c.1078G > T (p.G360C)	0.48
	E2 deletion	36.51
	c.1078 G > T (p.G360C)	1.66
	c.539 T > G (p.L180 W)	10.13
Gaucher	c.1448 T>C (p.L483P]/RecNciI^a^	101.11
MPS I	c.1093C > G (p.L365 V)/c.590-2A > C	396.83
MPS II	c.142C > T (p.R48C)	–
	c.1405C > G (p.P469A)	358.95
	c.779C > G (p.P260R)	350.60
MPS 3B	c.926A > G (p.Y309C)/c.848C > T(p.P283L)	896.56
	c.1693C > T (p.R565W)/c.1693C > T (p.R565W)	1139.71
	c.1226dupA (p.N409Kfs52)/c.1350G > T(p.Q450H)	596.03
ML	c.G1543A (p.A515T)/ c.637-6 T > G	319.13

The biomarkers are urinary Glc4 for Pompe (*N* < 12 mmol/mol Cre in newborns), plasma lysoGb3 for Fabry (*N* <  0.8 ng/mL), plasma lysoGb1 for GD (*N* < 3 ng/mL), total urine GAG (*N* < 312 mg GAGs/g Cre) for MPSs. ^a^patients on treatment

### Screening for Morquio disease

In total, 70 (0.09%) out of the 73,743 newborns had GALNS activity less than the screening cutoff, and a recall sample was requested. Twelve newborns still presented low GALNS activity, and a confirmatory follow-up was suggested. To better clarify the reason for low GALNS activity in these 12 samples, *GALNS* sequencing was performed. There were a total of 6 newborns having low GALNS activity and biallelic mutations (Table 3), with an incidence of one in 12,291 (95% CI: 5633-26,817). The novel mutation p.T286 M mutation was the most common variant (5 in 12 alleles, or 42%), while the allele frequency in the general population was 0.0036 (Additional file 3: Table S3). The other 6 babies, with low GALNS activity, had only one mutation and had normal total urine GAG. Thus were considered to be carriers. Among a total of 18 alleles found, three (p.A64I, p.P370S, p.P499L) were not reported previously *(*http://galns.mutdb.org/database)*.*

**Table 3:** Genotypes of newborns with positive MPS 4A screening

No	Genotype	DBS activity (% of normal mean)	Leukocyte activity (% of normal mean)	uKS
1	c.857C > T (p.T286 M) Ho*	0.38 (18)	19.48 (12)	1.47
2	c.857C > T (p.T286 M) Ho	0.24 (11)	19.81 (12)	0.87
3	c.1567 T > G (p.*523Eext*92)/c.1496C > T (p.P499L)	0.12 (6)	2.46 (2)	2.52
4	c.953 T > G(p.M318R) Ho	0.01 (0)	–	–
5	c.857C > T (p.T286 M)/ c.887C > T (p.A296V)	0.28 (13)	–	–
6	c.1019G > A (p.G340D)/ c.1108C > T (p.P370S)	0.31 (14)	12.33 (8)	2.45
7	c.953 T > G (p.M318R) He	0.22 (10)	16.44 (10)	0.60
8	c.857C > T (p.T286 M) He	0.28 (13)	15.44 (10)	0.73
9	c.190_191delinsAT (p.A64I) He	0.26 (12)	22.77 (14)	0.20
10	c.1127G > A (p.R376Q) Het	0.14 (6)	4.90 (3)	1.78
11	c.1177G > T (p.A393S) Het	0.39 (18)	14.25 (9)	1.26
12	c.1019G > A (p.G340D) He	0.26 (12)	10.51 (7)	1.48

Normal range: for DBS activity: normal mean 2.17 uM/hr.; for Leukocyte activity: 158.9 ± 82.8 nmol/mg Prot/17 h; for uKS: < 0.98 ng/ug Cre

*patients on treatment. One clinical case has leukocyte activity as 2.81 (2% of normal mean) and uKS as 3.73 ng/ug Cre before treatment

**Fig. 1:**
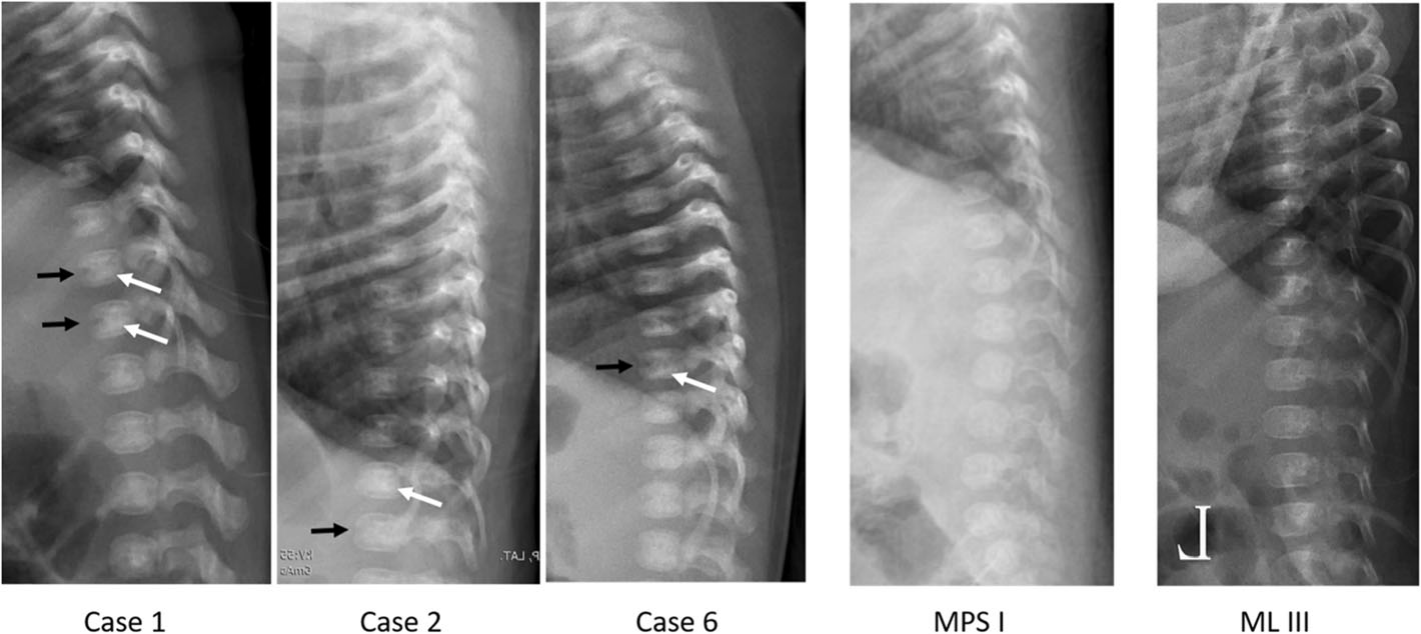
Thoracolumbar spine lateral views from three cases positive for MPS 4A screening (Case 1–3), one case positive for MPS I, and one case positive for ML III at ages 1–1.5 months. Abnormal findings in MPS 4A patients include incomplete fusion of the rostral and caudal halves of the vertebrae (black arrows), and ‘bone within bone’ appearance (white arrows); these findings are not seen in the MPS I or ML III patients. The images were rescaled and flipped for easy comparison

Spine X-rays and urine GAG analyses were obtained in three cases (patient 1, 2, and 6) at the age of 1–1.5 months (Fig. 1). In all 3 cases, fusion of the rostral and caudal halves of the vertebrae was incomplete (black arrows), and ‘bone within bone’ appearance (appearance of a lucent area within the outer aspect of the ossified vertebral body) was prominent (white arrows), suggesting a delay in vertebrae maturation in newborns with low GALNS activity. On the other hand, age-matched babies with IDUA deficiency and partial GALNS deficiency showed normal vertebrate maturation.

Patient 1, homozygous with the novel variant T286 M, had leukocyte GALNS activity of 19.48 nmol/mg Prot/17 h, 12% of the normal population mean. She was found to have urinary total GAG as 426.99 mg GAGs/g Cre (the 90th percentile at the age-matched control as 312) and keratan sulfate (KS) excretion as 1.47 ng/ug Cre (*N* <  0.98) at the time of diagnosis. Elosulfase alfa 2 mg/kg/week was initiated starting at 4 months of age. The follow-up X-ray up to 12 months of age still showed a delay in vertebrae maturation, but no anterior beaking of the vertebrae nor central pointing of the proximal part of metacarpal bones.

### Assay performance

We also measured the analytical range of the assays in our 8-plex and compared them to those measured for our original 4-plex assay. Results are summarized in Additional file 2: Table S2. The analytical range is defined as the ratio of assay response measured using a dried blood spot from a healthy control to that measured for the blank (filter paper only, no blood). The analytical range for the MPS I, Fabry, and Gaucher assays was found to be higher in the 8-plex assay compared to the 4-plex. This is likely due to the use of UPLC-MS/MS for the 8-plex compared to flow-injection-MS/MS. For the latter, in-source breakdown of the substrate to products leads to an increase in the assay response measured for the blank. However, with UPLC-MS/MS, substrate and product are separated during UPLC, and thus in-source breakdown does not increase the background as only the product signal eluting at the UPLC retention time of the product is integrated.

## Discussion

We report here the first prospective screen of an 8-plex LSDs UPLC-MS/MS assay that included screening for MPS 2, 3B, 4A and 6, plus the original potential of screening for Niemann-Pick A/B and Krabbe disease. This UPLC-MS/MS assay can be readily expanded to include other diseases such as type 2 Neuronal Ceroid Lipofuscinosis [1]. In addition, the multiplex assay enables us to simultaneously detect multiple sulfatase deficiency as this would be indicated if multiple sulfatases show low activity (MPS 2, MPS 4A and MPS 6 in our assay). Also, high activities of multiple lysosomal enzymes in dried blood spots are expected for patients with mucolipidosis. Indeed, we found one such patient in our study who had high activities of the MPS 2 and MPS 3B enzymes, which was confirmed by DNA analysis. Data for the live newborn screening of MPS 2 in Illinois [11] and a pilot for MPS 2, MPS 3B, MPS 4A, MPS 6, and MPS 7 using de-identified samples has recently been completed in Washington state [7] emphasize the advantage of this multiplex assay for LSD NBS.

Although the assay is highly accurate, we showed how a multi-tier approach for Pompe disease is very helpful to sort out affected by the relatively large number of pseudodeficiency samples we find in Taiwan. With this approach, we successfully identified only 6 at-risk babies using the first samples while minimizing the call-out of a large number of false positives (which is time consuming, expensive and stressful to families). In addition, using this 2-tier approach minimize the time of birth to the confirmatory diagnosis. We need only 2–3 days for cases met the critical cutoff and another 1–2 days for cases met the borderline cutoff. Therefore, the screened positive newborns could be referred to at the age of 6–9 days, and the one with infantile-onset Pompe disease even not met the critical cutoff could be treated by the age of 2 weeks.

We report here the results from the first large-scale, prospective MPS 4A newborn screening study. For the conservative purpose, we used 15% of the population mean GALNS activity as the cutoff. We found a surprisingly high incidence rate of 1 in 12, 291 (5633 to 26, 817). However, in our 6 newborns who tested positive for MPS 4A with biallelic *GALNS* variants, one displayed a GALNS activity of 0%. Therefore, the incidence of the potential severe MPS4A phenotype would be 1 in 73,743 (1 in 13,020 to 417,750), compatible with the previous clinical experience as 1 in 300,000 births in Taiwan [12]. One small scale newborn screening study involving 7415 samples revealed no babies with GALNS quantity below 15% of the normal population. In this study, the confirmed clinical patients had GALNS levels far below 5% of the normal population [13]. Enzymatic activity was not measured in this small pilot study, rather the quantity of GALNS protein was measured by an immunoassay. It is always possible that some MPS 4A patients have near-normal amounts of GALNS protein (i.e., mutations do not significant affect protein folding) but lack activity due to mutation of catalytically important amino acids. In our study, we directly measured GALNS enzymatic activity in dried blood spots. Our current study reveals that previous estimates of the incidence of MPS 4A in Taiwan may be underestimated, especially the mild phenotypes [14–16].

Three of the 6 GALNS deficiency babies were followed, and they all revealed a delay in vertebrae maturation, a finding was commonly seen in premature infants compared to full-term normal infants [17]. The deficiency of GALNS disrupts the normal development and maturation of cartilage and bone and subsequently gives rise to numerous structural anomalies of the spine [18]. Patients treated with ERT showed no statistically significant improvement in the height and growth rate [8, 19], probably due to delay in the initiation of treatment. In our newborns who are suspected to be affected with the attenuated form, only delay in maturation was observed. There were no other skeletal anomalies. The baby treated with ERT showed improvement of maturation 6 months after treatment. Whether the early initiation of enzyme replacement improves bone health remains to be further investigated. An individualized clinical follow-up plan is arguably the best option given the complex spectrum of symptoms.

## Conclusions

We report here that newborn screening for MPS 4A is feasible by measurement of the relevant enzymatic activity in dried blood spots together with enzymatic assays for 7 additional LSDs. The number of first-tier below cutoff samples was very low, 12 out of 73,743, leading to a manageable number of follow-up cases. Furthermore, 6 of the 12 screen positives were found to have biallelic GALNS mutations in trans. Nevertheless, there remains the need for careful patient follow-up in cases detected by newborn screening, where later-onset symptoms are predicted, and the full understanding of pseudodeficiencies is still needed. Thus, the long-term follow-up of these infants will be essential to understand the phenotypes detected by newborn screening fully. The impact of early treatment of MPS 4A following newborn screening also remains to be studied.

## Supplementary information

**Additional file 1: Table S1.** Enzyme activity or activity ratio cutoffs for newborn screening

**Additional file 2: Table S2.** Analytical range values (activity measured in the DBS divided by that measured in the no DBS-blood blank) obtained using a 3mm punch of a DBS made from a healthy adult.

**Additional file3: Table S3.** *GALNS* variants found in this study

**Additional file 4: Figure S1.** The chromatogram and MRM transitions of products and internal standards.

## Data Availability

All data generated or analyzed during this study are available from the corresponding author on reasonable request.
