# Supplementary material for: Newborn screening for Morquio disease and other lysosomal storage diseases: results from the 8-plex assay for 70,000 newborns
Source: Orphanet J Rare Dis. 2020 Feb 3;15:38. doi: 10.1186/s13023-020-1322-z (PMC6998831; doi:10.1186/s13023-020-1322-z)
Supplement: Supplementary file 1 — Additional file 1: Table S1. Enzyme activity or activity ratio cutoffs for newborn screening [file 13023_2020_1322_MOESM1_ESM.docx]

**Supplement online**

Table S1. Enzyme activity or activity ratio cutoffs for newborn screening

| Condition | Enzyme | cutoff |
| --- | --- | --- |
| Pompe | acid α-glucosidase (GAA) | GAA ≤ 1.2 µM/h (~15% of normal mean) or ABG/GAA ≥ 10 |
| Fabry | acid α-galactosidase (GLA) | GLA ≤ 1.2 µM/h (~15% of normal mean) or ABG/GLA ≥ 15 |
| Gaucher | acid β-glucocerebrosidase (ABG) | ABG ≤ 2.5 µM/h (~20% of normal mean) |
| MPS I | α-L-iduronidase (IDUA) | IDUA ≤ 2.2 µM/h (~30% of normal mean) |
| MPS II | iduronate-2-sulfatase(I2S) | I2S ≤ 5% of normal mean |
| MPS 3B | α-N-acetylglucosaminidase (NAGLU) | NAGLU ≤ 15% of normal mean |
| MPS 4A | N-acetylgalactosamine-6-sulfatase (GALNS) | GALNS ≤ 15% of normal mean |
| MPS 6 | N-acetylgalactosamine-4-sulfatase (ARSB) | ARSB ≤ 15% of normal mean |
