# Supplementary material for: Newborn screening for Morquio disease and other lysosomal storage diseases: results from the 8-plex assay for 70,000 newborns
Source: Orphanet J Rare Dis. 2020 Feb 3;15:38. doi: 10.1186/s13023-020-1322-z (PMC6998831; doi:10.1186/s13023-020-1322-z)
Supplement: Supplementary file 2 — Additional file 2: Table S2. Analytical range values (activity measured in the DBS divided by that measured in the no DBS-blood blank) obtained using a 3mm punch of a DBS made from a healthy adult. [file 13023_2020_1322_MOESM2_ESM.docx]

Table S2. Analytical range values (activity measured in the DBS divided by that measured in the no DBS-blood blank) obtained using a 3mm punch of a DBS made from a healthy adult.

|  | **FIA-MS/MS 4-Plex** | **LC-MS/MS 8-Plex** |
| --- | --- | --- |
| Pompe | 851 | 792 |
| Fabry | 118 | 307 |
| Gaucher | 101 | 142 |
| MPS I | 78 | 439 |
| MPS II |  | 537 |
| MPS 3B |  | 887 |
| MPS 4A |  | 93 |
| MPS 6 |  | 506 |

1Activities are the mean of triplicate runs.

2The analytical range is the activity with 3 DBS punch divided by blank activity with filter paper punch (no blood).
