# Supplementary material for: Newborn screening for Morquio disease and other lysosomal storage diseases: results from the 8-plex assay for 70,000 newborns
Source: Orphanet J Rare Dis. 2020 Feb 3;15:38. doi: 10.1186/s13023-020-1322-z (PMC6998831; doi:10.1186/s13023-020-1322-z)
Supplement: Supplementary file 3 — Additional file3: Table S3. GALNS variants found in this study [file 13023_2020_1322_MOESM3_ESM.docx]

Table S3. *GALNS* variants found in this study

| Amino acid changes | dbSNP | Clinvar | Allele frequency from Taiwan BioBank |
| --- | --- | --- | --- |
| T286M | rs137927658 | **Uncertain significance​** | 0.00363 |
| A296V | rs200371805 | Not report | 0.00396 |
| R376Q | rs150734270 | **Uncertain significance​** | 0.00247 |
| A393S | rs2303269 | **Benign/Likely benign​** | 0.05275 |
| G340D | rs267606838 | **Pathogenic** | NF |
| P370S | rs749891007 | Not report | NF |
| M318R | rs746756997 | **Pathogenic** | NF |
| P499L | rs542835085 | Not report | NF |
| c.190_191delinsAT(p.A64I) | rs769112202 | Not report | NF |
|  | rs527906032 | Not report | NF |

Taiwan BioBank data is searched from Taiwan View (<https://taiwanview.twbiobank.org.tw/index>). NF: not found.
