# Supplementary material for: Newborn screening for Morquio disease and other lysosomal storage diseases: results from the 8-plex assay for 70,000 newborns
Source: Orphanet J Rare Dis. 2020 Feb 3;15:38. doi: 10.1186/s13023-020-1322-z (PMC6998831; doi:10.1186/s13023-020-1322-z)
Supplement: Supplementary file 4 — Additional file 4: Figure S1. The chromatogram and MRM transitions of products and internal standards. [file 13023_2020_1322_MOESM4_ESM.docx]

Figure S1: The chromatogram and MRM transitions of products and internal standards.
